# Bacterial 3′UTRs: A Useful Resource in Post-transcriptional Regulation

**DOI:** 10.3389/fmolb.2020.617633

**Published:** 2021-01-08

**Authors:** Pilar Menendez-Gil, Alejandro Toledo-Arana

**Affiliations:** Instituto de Agrobiotecnología (IdAB), Consejo Superior de Investigaciones Científicas (CSIC) - Gobierno de Navarra, Navarra, Spain

**Keywords:** 3′UTR, bacteria, gene expression regulation, RNA-binding proteins (RBPs), ncRNAs (non-coding RNAs), regulatory small RNAs (sRNAs), post-transcription regulation

## Abstract

Bacterial messenger RNAs (mRNAs) are composed of 5′ and 3′ untranslated regions (UTRs) that flank the coding sequences (CDSs). In eukaryotes, 3′UTRs play key roles in post-transcriptional regulatory mechanisms. Shortening or deregulation of these regions is associated with diseases such as cancer and metabolic disorders. Comparatively, little is known about the functions of 3′UTRs in bacteria. Over the past few years, 3′UTRs have emerged as important players in the regulation of relevant bacterial processes such as virulence, iron metabolism, and biofilm formation. This MiniReview is an update for the different 3′UTR-mediated mechanisms that regulate gene expression in bacteria. Some of these include 3′UTRs that interact with the 5′UTR of the same transcript to modulate translation, 3′UTRs that are targeted by specific ribonucleases, RNA-binding proteins and small RNAs (sRNAs), and 3′UTRs that act as reservoirs of *trans-*acting sRNAs, among others. In addition, recent findings regarding a differential evolution of bacterial 3′UTRs and its impact in the species-specific expression of orthologous genes are also discussed.

## Introduction

Post-transcriptional regulation in bacteria has emerged as an essential layer to tightly control gene expression. Different regulatory elements are involved in the modulation of messenger RNA (mRNA) elongation, stability, and translation. Genomes encode a large variety of RNA-binding proteins (RBPs) and regulatory RNAs that target mRNAs and modify their expression in diverse ways. Among RBPs, ribonucleases (RNases) play key roles in both the maturation and degradation of mRNAs, which result in improved translation and disposal of no longer required transcripts, respectively. Other RBPs such as RNA chaperones, RNA helicases, and RNA methyltransferases modify the susceptibility of transcripts to RNases and the accessibility of mRNAs to ribosomes. Some of these RBPs also assist in the interaction between regulatory RNAs and their targeting mRNAs or regulate the formation of transcriptional terminator/anti-terminator structures (Van Assche et al., 2015; Holmqvist and Vogel, 2018; Woodson et al., 2018). Regarding regulatory RNAs, two large categories can be distinguished: small RNAs (sRNAs) and *cis*-encoded antisense RNAs (asRNAs). sRNAs target mRNAs in *trans* and are often encoded in a different genomic location from their targets. The nucleotide pairing between a sRNA and its target mRNA is usually imperfect and it often requires the assistance of RNA chaperones (Wagner and Romby, 2015; Dutcher and Raghavan, 2018). In contrast, asRNAs are encoded in the opposite DNA strand of their targets and thus their interaction produces a perfect pairing between both RNA molecules. The resultant RNA duplexes can be processed by double-stranded endoribonucleases such as RNase III (Georg and Hess, 2011; Toledo-Arana and Lasa, 2020). Besides these regulatory RNAs, the untranslated regions (UTRs) of the mRNAs may contain regulatory elements, which modulate the expression of their own mRNAs in different ways. Monocistronic and polycistronic mRNAs are comprised of two UTRs that flank the coding sequence(s) (CDSs), the 5′- and the 3′UTR, respectively. A major breakthrough in the discovery of functional bacterial UTRs was the genome-wide transcriptomic mapping, which showed that their lengths were greater than previously anticipated, suggesting that they could act as a reservoir of additional regulatory elements (Toledo-Arana et al., 2009). This was more evident for the 5′UTRs, which have been well-known for including riboswitches and thermosensors that control the expression of their downstream CDSs (Loh et al., 2018; Pavlova et al., 2019). In addition, several examples of long 5′UTRs have been described to overlap the mRNAs encoded in the opposite DNA strand (Toledo-Arana et al., 2009; Sesto et al., 2012; Toledo-Arana and Lasa, 2020). In contrast, little is known about the 3′UTRs functions, probably because post-transcriptional regulation studies mainly focused on sRNAs and 5′UTRs. Additionally, the first RNA-sequencing techniques did not map the 3′ ends, leaving in the dark relevant information about them. However, recent discoveries have brought to light that 3′UTRs can play relevant roles by tightly modulating gene expression in bacteria through diverse mechanisms.

In eukaryotes, 3′UTRs are far longer than 5′UTRs and they constitute key components of the overall post-transcriptional regulation (Pesole et al., 2002; Mazumder et al., 2003). Eukaryotic 3′UTRs possess a wide variety of regulatory motifs that are recognized by microRNAs (miRNAs) and RBPs to control mRNA stability, localization, and translation (Mazumder et al., 2003; Mayr, 2017). The AU-rich and GU-rich elements (AREs and GREs, respectively) constitute good examples of this as their sequences are recognized by proteins that favor mRNA degradation (Halees et al., 2008, 2011; Vlasova et al., 2008; von Roretz et al., 2011). The polyA tail (a stretch of as located in the 3′ end of the mRNA) is another element that can determine mRNA stability and, as a result, protein levels. Alternative 3′UTRs not only affect mRNA stability and translation but also control mRNA localization (Tushev et al., 2018). There are cases in which a poly(A)-binding protein (PABP) can cause a positive effect on their mRNA targets by promoting translation (Mayr, 2017). Once the PABP binds to the poly(A), it interacts with translation factor eIF4G which, in turn, binds the cap-binding protein eIF4E that is bound to the capped 5′ end. This generates an mRNA circularization that promotes the engagement of terminating ribosomes to a new round of translation of the same mRNA, thus, enhancing protein synthesis (Wells et al., 1998; Alekhina et al., 2020).

Interestingly, the length of the eukaryotic 3′UTRs varies according to the protein encoded in the mRNA. Frequently, mRNAs that encode housekeeping proteins that are highly expressed possess single 3′UTRs. In contrast, mRNAs expressing regulatory proteins, which are tightly regulated, contain multiple alternative 3′UTRs (Lianoglou et al., 2013). Moreover, there is a number of examples of mRNAs that produce alternative 3′UTRs depending on the cell tissues where they will be expressed (Mayr, 2016). Alternative 3′UTRs can modulate, among others, the localization of membrane proteins in human cells. During mRNA translation, 3′UTRs work as scaffolds that facilitate the binding of proteins to the nascent protein, directing their transport or function (Berkovits and Mayr, 2015). Deregulation or shortening of 3′UTRs is associated with diverse diseases such as cancer or metabolic disorders (Khabar, 2010; Mayr, 2017). It is noteworthy that there is a direct correlation between the 3′UTR length and the evolutionary age and complexity of organisms, with the longest 3′UTRs being found in the human genome. Contrary to this, 5′UTRs have preserved an almost constant length through the course of evolution. This 3′UTR evolutionary lengthening suggests that 3′UTR-mediated post-transcriptional regulation must have played a significant role in generating functional differences between species (Mazumder et al., 2003; Mayr, 2017).

Over the past few years, several of the 3′UTR-mediated regulatory mechanisms that were initially described for eukaryotes have also been discovered in bacteria. In this review, the current known mechanisms to control gene expression in bacteria through 3′UTRs are addressed. In addition, the differential evolution found within this bacterial mRNA region and the differences it has created between mRNAs that encode orthologous proteins is also discussed.

## Identification of 3′UTRs in Bacteria

The 3′UTR is the section of nucleotides found between the translation stop codon and the transcription termination site in an mRNA molecule. Before the transcriptomic era, the efforts in understanding transcript boundaries were biased toward the 5′ end. Several reasons could explain this bias. On the one hand, the 5′ end was required for identifying promoter sequences as well as putative transcriptional regulatory regions and RNA secondary structures that contributed to gene expression control. On the other hand, the lack of the 3′ poly(A) tail in bacterial mRNAs prevented a direct reverse transcription priming from the 3′ end, rendering their mapping challenging. The 3′ end of particular transcripts was identified by classical S1 nuclease mapping [protocol updated in Sambrook and Russell (2006)] and, later on, through a modified 5′/3′ RACE method (rapid amplification of cDNA ends) that simultaneously mapped 5′ and 3′ ends of RNA ligase-circularized RNAs (Britton et al., 2007). At the same time, it was a general belief that the 3′UTRs would not play any major roles in bacterial gene expression control and that their functions were restricted to transcript termination and protection of the mRNAs from RNases. This perception quickly changed with the accomplishment of the first genome-wide transcriptomic maps. Although the resolution of these transcriptomes was restricted by the technologies used at that time (e.g., high-resolution tiling arrays), they started to unveil how 3′UTRs could play more impactful roles than initially anticipated (Rasmussen et al., 2009; Toledo-Arana et al., 2009; Broeke-Smits et al., 2010). Later on, thanks to the development of high-throughput stranded RNA sequencing (RNA-seq), the identification of transcript boundaries was significantly simplified. RNA-seq confirmed the relevance of 3′UTRs, showing that they encode a wide variety of regulatory elements (for details see following sections). Nowadays, genome-wide maps of 3′ ends at single-nucleotide resolution of bacteria grown in different conditions can be obtained by Term-seq, which directly sequences exposed RNA 3′ ends (Dar et al., 2016). This has been recently used to determine and compare the 3′ ends from bacterial models such as *Escherichia coli, Bacillus subtilis, Listeria monocytogenes, Enterococcus faecalis*, and *Staphylococcus aureus* (Dar et al., 2016; Dar and Sorek, 2018b; Menendez-Gil et al., 2020). In addition, other RNA-seq based techniques that were envisioned for the identification of RNA-RNA and RNA-protein interactions and ribonuclease processing sites have also provided the scientific community with novel putative functional 3′UTR candidates. For example, the RIL-seq (RNA interaction by ligation and sequencing), RIP-seq (RNA immunoprecipitation followed by RNA sequencing), TIER-seq (transiently-inactivating-an-endoribonuclease-followed-by-RNA-seq) and CLASH (UV cross-linking, ligation, and sequencing of hybrids) methods have revealed dozens of sRNAs candidates originated from 3′UTRs of several enterobacterial species (Holmqvist et al., 2016, 2018; Melamed et al., 2016, 2019; Chao et al., 2017; Hoyos et al., 2020; Huber et al., 2020; Iosub et al., 2020). Combining these technologies with those dedicated to 5′ and 3′ end mapping (Sharma et al., 2010; Dar et al., 2016) would complete the bacterial transcriptomic landscapes and their RNA-RNA and RNA-protein network interactions, which are essential for understanding post-transcriptional regulatory mechanisms, including those involving 3′UTRs.

## Bacterial 3′UTRs Are Longer Than Previously Expected

The 3′UTR usually harbors the transcriptional termination signal, which could be an intrinsic terminator or a Rho utilization (*rut)* site (Peters et al., 2011). The intrinsic terminator consists of a hairpin structure followed by a U-tract that promotes transcript release from the RNA polymerase (RNAP) (Rho-independent termination), while the *rut* site recruits the Rho protein to produce a Rho-dependent termination. When Rho interacts with *rut*, its ATPase activity is triggered, providing it with the necessary energy to translocate along the mRNA. Transcription termination occurs thanks to the Rho helicase activity that causes a dissociation of the RNAP from the transcript (Peters et al., 2011). Rho is widespread in bacteria, however, Rho-dependent termination is more common in Gram-negative bacteria whereas in Gram-positive bacteria intrinsic terminators seem to be the norm (Ciampi, 2006). The average length of an intrinsic terminator was estimated in *S. aureus* to be around 30 nucleotides (Ruiz de Los Mozos et al., 2013). Therefore, a 3′UTR of 40-50 nt long would be sufficient to allocate a functional intrinsic terminator. However, the mapping of 3′ ends by genome-wide RNA sequencing methods in bacteria revealed that several 3′UTRs were much longer than previously anticipated (Broeke-Smits et al., 2010; Ruiz de Los Mozos et al., 2013; Dar et al., 2016; Dar and Sorek, 2018b). In *S. aureus*, more than 30% of the mapped 3′UTRs showed lengths above 100 nt. This was a strong evidence indicating that 3′UTRs could allocate additional regulatory elements (Ruiz de Los Mozos et al., 2013).

Long 3′UTRs are generated when the transcriptional terminator signals are located far away from their corresponding translational stop codons. In addition, since bacterial transcriptional termination mechanisms are not always effective in dissociating the RNAP from the nascent transcript, alternative read-through-mediated transcripts may also be generated. In Rho-independent termination, the level of read-through is proportional to the strength of the intrinsic terminator. The effectiveness of these terminators is favored by several factors such as the U-tract located immediately downstream of the hairpin structure, which promotes disassociation from the RNAP (Cambray et al., 2013; Chen et al., 2013). Other elements involve the enrichment of GC nucleotides at the stem of the hairpin, which favors its folding (Chen et al., 2013). Conversely, certain RBPs can induce transcription elongation by forcing the RNA polymerase to ignore the intrinsic transcriptional termination signal and thus generate longer alternative transcripts (Phadtare et al., 2002; Goodson et al., 2017; Goodson and Winkler, 2018).

Regarding Rho-dependent termination, several investigations depict Rho as a key factor that controls pervasive read-through transcription in bacteria. Deletion of the *rho* gene or inhibition of Rho activity using bicyclomycin causes transcriptional read-through (Nicolas et al., 2012; Mäder et al., 2016; Bidnenko et al., 2017; Dar and Sorek, 2018b).

**Figure 1:**
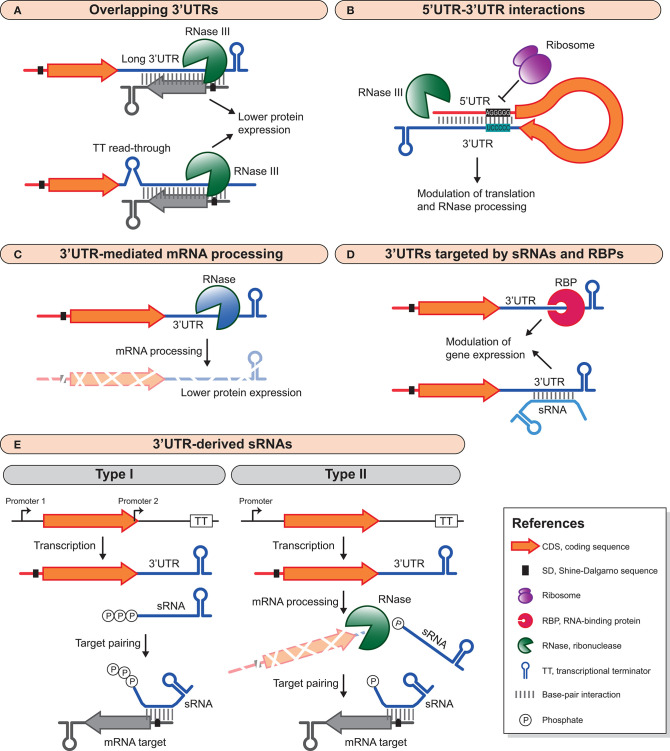
Different 3′UTR-mediated regulatory mechanisms in bacteria. **(A)** Long 3′UTRs and transcriptional read-through can produce overlapping 3′UTRs that modulate the expression of convergent genes. These overlapping double-stranded RNA regions are processed by RNase III, which ultimately decreases protein expression. **(B)** 3′UTRs can interact with the 5′UTR of the same mRNA to modulate mRNA stability and translation. This interaction can inhibit translation and recruit RNases for mRNA degradation, or it can promote mRNA stability by impairing RNase processing. **(C)** RNases can specifically target 3′UTRs to process mRNAs and modify their half-life and protein expression yield. **(D)** sRNAs and RBPs can target 3′UTRs to modulate mRNA expression. This interaction can be both positive, by blocking RNase processing and enhancing mRNA stability, or negative by promoting inhibition of translation and RNase processing. **(E)** 3′UTRs are also reservoirs of *trans*-acting sRNAs than can be generated by an internal promoter within or immediately downstream of the CDS (type I) or by mRNA processing (type II). For examples, see text and Table 1.

Since bacterial genomes are compact and the distance between CDSs is often short, when transcriptional terminator signals between convergent genes are missing and/or transcriptional read-through occurs, long antisense overlapping 3′UTRs are produced (Hernández et al., 2006; Toledo-Arana et al., 2009; Arnvig et al., 2011; Lasa et al., 2011; Nicolas et al., 2012; Moody et al., 2013; Mäder et al., 2016; Stazic and Voss, 2016; Bidnenko et al., 2017; Huang et al., 2020; Toledo-Arana and Lasa, 2020) (Figure 1A). In addition, more complex transcriptional organizations like operons containing a gene(s) that is transcribed in the opposite direction to the rest of the operon are also known for generating long overlapping transcripts (Lasa et al., 2011; Sáenz-Lahoya et al., 2019). The outcome is usually the development of large double-stranded RNA regions that are processed by RNase III in both Gram-positive and Gram-negative bacteria (Lasa et al., 2011, 2012; Gatewood et al., 2012; Lioliou et al., 2012; Lybecker et al., 2014; Huang et al., 2020). Interestingly, overlapping 3′UTRs are widely distributed in bacteria and constitute an abundant source of antisense RNAs (Arnvig et al., 2011; Lasa et al., 2011; Ruiz de Los Mozos et al., 2013). These antisense mechanisms, which often involve mRNAs encoding proteins with opposing functions and whose expression is mutually regulated, are examples of the *excludon* concept (Sesto et al., 2012).

In *B. subtilis*, Rho inhibition impairs the correct switch between motility, biofilm formation and sporulation. Rather than considering this as a pleiotropic effect, Bidnenko et al. demonstrated that the control of these opposite phenotypes was determined by a specific architecture of the Rho-controlled transcriptome, which involved several overlapping 3′UTRs. The different required elements appeared to be organized for the simultaneous activation of one phenotype while repressing the alternative ones and, thus, providing the *excludon* with a biological meaning (Bidnenko et al., 2017). It is noteworthy that Rho-dependent termination is also susceptible to regulation by sRNAs (Bossi et al., 2012; Wang et al., 2015; Chen et al., 2019).

Finally, there is knowledge of an additional complex genomic architecture surrounding riboswitches that could further increase the source of long 3′UTRs. Riboswitches are regulatory elements located at the 5′UTRs that control the expression of the downstream genes in function of the concentration of a specific metabolite. The riboswitch sensor induces a conformational change in the expression platform upon binding of such metabolite, leading to changes in the transcript elongation and/or the translation of downstream genes (Serganov and Nudler, 2013). Interestingly, riboswitches that work as attenuators can also control the transcript termination of upstream genes. This occurs when genes located upstream of riboswitches lack a transcriptional termination signal. Therefore, transcription of upstream genes may terminate or continue depending on the riboswitch configuration. If the riboswitch is in a transcriptional OFF-configuration, the transcription of upstream genes stops, as these riboswitches form a hairpin that works as an intrinsic terminator. The regenerated transcript would contain a long 3′UTR including the riboswitch sequence. In contrast, if the riboswitch is in an ON-configuration, the result is a long polycistronic transcript that includes the initial gene plus the riboswitch and the downstream genes. This transcriptomic configuration was initially described in *L. monocytogenes*, but it is present in several bacterial species (Toledo-Arana et al., 2009; Ruiz de Los Mozos et al., 2013). The consequences of this transcriptomic architecture on the expression and function of the genes involved still needs to be elucidated.

The evidence of different regulatory factors that modulate both intrinsic and Rho-dependent transcriptional termination mechanisms opens the possibility to transcriptional read-through events as an additional regulatory layer. This would imply that bacterial chromosomes contain larger transcriptionally-connected chromosomic regions that go beyond the classical operon configurations known so far (Toledo-Arana and Lasa, 2020).

## 3′UTRs as Modulators of Gene Expression

Besides the reciprocal antisense regulation exerted by the 3′UTR-overlapping mRNAs explained above, 3′UTRs can modulate gene expression in many other ways (Figure 1). Some 3′UTRs generate small regulatory RNAs that act in *trans* by targeting mRNAs, while others modulate the expression of their own mRNA. Additionally, 3′UTRs may be targeted by sRNAs and/or RBPs to modify 3′UTR function.

### 3′UTRs That Interact With the 5′UTRs of the Same mRNA Molecule

The first example that showed a bacterial 3′UTR modulating the expression of the protein encoded in its own mRNA was the paradigmatic RNAIII of *S. aureus* described by Balaban and Novick (Balaban and Novick, 1995). RNAIII is a dual-function RNA that encodes the δ-hemolysin and a regulatory *trans*-acting RNA that base pairs with several virulence-related mRNAs to modulate their expression (Bronesky et al., 2016; Raina et al., 2018). It was shown that translation of δ-hemolysin occurred one hour after transcription of RNAIII. This delay was non-existent when the 3′UTR was deleted. *In-silico* RNA structural analyses predicted a 5′UTR-3′UTR interaction as a plausible cause for said post-transcriptional regulation (Balaban and Novick, 1995). The long-range 5′UTR-3′UTR interaction was later on validated by chemical and enzymatic probing analyses, which demonstrated the base-pairing of residues A24-G30 and U452-458 (Benito et al., 2000). Whether this 5′UTR-3′UTR interaction directly modulates δ-hemolysin translation remains to be demonstrated by site-directed mutagenesis.

Several years passed until novel functional 3′UTRs were characterized. Our group unveiled that the 3′UTR of the *icaR* mRNA in *S. aureus* interacted with the ribosome binding site (RBS) of its own mRNA through a UCCCC sequence that behaved as an anti-RBS motif (Figure 1B). This 5′UTR-3′UTR interaction inhibited translation initiation of the IcaR protein and produced a double-stranded RNA substrate that was cleaved by RNase III, leading to mRNA degradation. IcaR is the repressor of the *ica* operon, which encodes the main exopolysaccharidic compound (PIA-PNAG) of the *S. aureus* biofilms. Therefore, the *icaR* 5′UTR-3′UTR interaction favored biofilm formation (Ruiz de Los Mozos et al., 2013). A few years later, a 14 nt interaction between the 3′UTR and the 5′UTR of the *hbs* mRNA in *B. subtilis* was uncovered. This 5′-3′UTR interaction protected the *hbs* mRNA from RNase Y cleavage at the 5′UTR (Braun et al., 2017). The regulatory 5′-3′UTRs interactions are in agreement with a recent study in *B. subtilis* that shows how transcription and translation are fundamentally disjointed (Johnson et al., 2020). Faster RNAPs in combination with highly structured 5′UTRs would favor the production of ribosome-free mRNAs. These would likely be more susceptible to post-transcriptional regulatory processes since they would not be interfered by translating ribosomes (Johnson et al., 2020). Whether this type of 5′-3′UTR interaction mechanism applies to the regulation of other bacterial genes requires further investigations.

### 3′UTR-Mediated mRNA Processing

Several examples of 3′UTRs that are involved in mRNA processing have recently been revealed for different bacterial species. 3′UTRs can harbor RNase cleavage sites that, upon processing, modify the stability and turnover of mRNAs (Figure 1C). It has been demonstrated that most of these bacterial 3′UTRs act as independent modules. Cloning the 3′UTRs downstream of the *gfp or lacZ* reporter genes proved that the 3′UTRs retained their functional capabilities since they were still being targeted by the same RNases. Moreover, the regulatory effect on the expression of the upstream reporter genes was similar to the one observed for their original CDSs (Maeda and Wachi, 2012; López-Garrido et al., 2014; Zhu et al., 2015; Zhao et al., 2018; Menendez-Gil et al., 2020).

In *S. aureus*, the *rpiRc* and *ftnA* 3′UTRs are able to modify their mRNAs stability (Menendez-Gil et al., 2020). RpiRc is a transcriptional repressor of the Agr quorum sensing system and therefore a virulence regulator (Zhu et al., 2011; Balasubramanian et al., 2016; Gaupp et al., 2016), while FtnA is an iron storage protein that modulates intracellular iron availability (Morrissey et al., 2004; Zühlke et al., 2016). Deletion of both 3′UTRs increase the mRNA and protein levels of their respective genes, indicating that the expression of these proteins may be modulated by still unidentified ribonucleases (Menendez-Gil et al., 2020).

In *Yersinia pestis*, several long 3′UTRs with AU-rich motifs induce mRNA degradation (Zhu et al., 2015; Zhao et al., 2018). Among them, the *hmsT* 3′UTR seems of relevance due to its ability to modulated c-di-GMP metabolism and biofilm formation (Zhu et al., 2015). Note that AREs are key *cis*-acting factors involved in the regulation of many important cellular processes in eukaryotes, such as stress response, inflammation, immuno-activation, apoptosis, and carcinogenesis. AREs are located in the 3′UTRs of short-lived mRNAs, which function as a signal for rapid degradation (Bakheet et al., 2006; Halees et al., 2008). Multiple RBPs regulate the transport, stability, and translation of eukaryotic mRNAs containing AREs (García-Mauriño et al., 2017). Some of the *Y. pestis* AU-rich 3′UTRs behave in a similar way since they are specifically targeted by the polynucleotide phosphorylase (PNPase), a 3′-5′ exoribonuclease, and might be also acting as inductors of rapid mRNA degradation (Zhao et al., 2018).

Other examples of mRNAs containing AU-rich motifs in their 3′UTRs that promote mRNA processing are found in *Salmonella* and *Corynebacterim glutamicum* (Maeda and Wachi, 2012; López-Garrido et al., 2014). PNPase and RNase E target the 3′UTR of the *hilD* mRNA, which encodes one of the main transcriptional activators of *Salmonella* pathogenicity island 1 (SPI-1) (López-Garrido et al., 2014). Similarly, RNase E/G processes the *aceA* mRNA of *C. glutamicum*. This mRNA encodes the isocitrate lyase protein, an enzyme of the glyoxylate cycle (Maeda and Wachi, 2012). Since PNPase activity is inhibited by hairpin structures such as those found in intrinsic terminators (Hui et al., 2014; Dar and Sorek, 2018a), it is possible that AU-rich 3′UTRs are initially cleaved by RNase E providing an accessible 3′ end for the action of PNPase. Note that RNase E is an endoribonuclease that preferentially targets AU-rich regions in single-stranded RNAs (McDowall et al., 1994). Chao et al. demonstrated that RNase E sites were enriched around mRNA stop codons. The RNase E cleavage eliminates 3′ end protective hairpin structures, rendering processed 3′ ends accessible to degradation by 3′/5′ exoribonucleases such as PNPase (Chao et al., 2017; Dar and Sorek, 2018a). This observation has been recently confirmed in *Streptococcus pyogenes*, where RNase Y-processed 3′ ends are subsequently trimmed by PNPase and YhaM. RNase Y is the functional RNase E ortholog in Gram-positive bacteria (Broglia et al., 2020).

### 3′UTRs That Are Targeted by RBPs and sRNAs

The 3′UTR-mediated mRNA processing can be inhibited by RBPs (Figure 1D). For example, the expression of the *E. coli* aconitase (*acnB*) mRNA is autoregulated by its own protein. AcnB is an enzyme of the TCA (tricarboxylic acid) cycle that uses iron as a cofactor. However, upon iron starvation it becomes an autoregulatory RBP (apo-AcnB). The apo-AcnB protein binds its own mRNA at a stem-loop located in the 3′UTR, which is in close proximity to an RNase E cleavage site, preventing the *acnB* mRNA degradation by RNase E (Benjamin and Massé, 2014). Likewise, global regulatory RNA chaperones including Hfq, CsrA and ProQ also bind 3′UTRs (Holmqvist et al., 2016, 2018; Potts et al., 2017). Among them, ProQ targets secondary structures of 3′UTRs in *Salmonella* and *E. coli* to protect their mRNAs from RNases. Such is the case of the *cspE* mRNA, which is recognized by ProQ to prevent RNase II cleavage (Holmqvist et al., 2018). Hfq has preference for 3′UTRs that produce regulatory RNAs (Holmqvist et al., 2016). In comparison to Hfq and ProQ, the number of 3′UTRs recognized by CsrA is much lower and its regulatory role over this region, if any, remains unknown (Potts et al., 2017).

Eukaryotic 3′UTRs are targeted by microRNAs to modulate mRNA expression (Krol et al., 2010; Saliminejad et al., 2019). Recently, bacterial 3′UTRs have been also shown to be targeted by *trans*-acting regulatory RNAs (El-Mouali et al., 2018; Bronesky et al., 2019) (Figure 1D). In *S. aureus*, the RsaI sRNA binds to the 3′UTR of the *icaR* mRNA, modulating the PIA-PNAG exopolysaccharide biosynthesis. Interestingly, the catabolite control protein A (CcpA) represses RsaI expression when glucose is available in the culture medium. Therefore, the RsaI-*icaR* 3′UTR interaction links the glucose metabolism with the biofilm formation process (Bronesky et al., 2019). Since RsaI binds to a different region to that of the UCCCC motif (required for the *icaR* 5′UTR-3′UTR interaction), it is speculated that RsaI might help stabilizing the circularization of the mRNA and, ultimately, inhibit IcaR translation (Bronesky et al., 2019). In *Salmonella*, the *trans*-acting sRNA Spot 42 connects metabolism and virulence by interacting with the long 3′UTR of the *hilD* mRNA (El-Mouali et al., 2018). Spot 42 is repressed by the cAMP-activated global transcriptional regulator CRP. When environmental and/or physiological signals disrupt the CRP-dependent Spot 42 repression, Spot 42 becomes available to bind to the *hilD* 3′UTR in a Hfq-dependent manner. Spot 42 binding activates HilD protein expression and thereby induces the expression of HilD-dependent virulence genes (López-Garrido et al., 2014; El-Mouali et al., 2018; El-Mouali and Balsalobre, 2019).

### 3′UTRs That Produce *trans*-Acting sRNAs

3′UTRs have also emerged as reservoirs of *trans*-acting sRNAs (Kawano et al., 2005; Chao et al., 2012; Miyakoshi et al., 2015b). 3′UTR-derived sRNAs can be generated either by the presence of an internal promoter located within or immediately downstream of the CDS (type I) or by the processing of an mRNA transcript at the 3′UTR (type II). Therefore, type I sRNAs carry a triphosphate at their 5′ ends while type II sRNAs exhibit monophosphate 5′ ends (Miyakoshi et al., 2015b) (Figure 1E).

Although 3′UTR-derived sRNAs have been described in several bacterial species, most of them have been characterized in Gram-negative bacteria (Table 1). Interestingly, type II sRNAs are more abundant than type I, highlighting the relevance of specific RNase cleavage sites present within the 3′UTRs. The biogenesis of many type II 3′UTR-derived sRNAs depends on the mRNA cleavage by RNase E, whose target sites are enriched around the mRNA stop codon (Chao et al., 2017). Table 1 summarizes relevant bacterial 3′UTR-derived sRNA examples. The sRNA targets and their physiological roles are also indicated in this table. These examples suggest that 3′UTR-derived sRNAs are widely distributed in bacteria and that they control a broad variety of biological processes. For example, the type I MicL sRNA modulates envelope stress by repressing the synthesis of Lpp, a major outer membrane lipoprotein in *E. coli* (Guo et al., 2014), while DapZ and MicX control the expression of the major ABC transporters in *Salmonella* and *V. cholerae*, respectively (Davis and Waldor, 2007; Chao et al., 2012). This is further exemplified by the type II SorX, RsaC and s-SodF sRNAs that participate in the oxidative stress response of *Rhodobacter sphaeroides, S. aureus* and *Streptomyces coelicolor*, respectively (Kim et al., 2014; Peng et al., 2016; Lalaouna et al., 2019). Besides, 3′UTR-derived sRNAs also participate in autoregulating the expression of the genes encoded in the same mRNAs that generate them. For example, the *V. cholerae* OppZ and CarZ sRNAs, which originate from the *oppABCDF* and *carAB* operons upon RNase E processing, respectively, base-pair with their own transcripts, leading to translation initiation inhibition and followed by Rho-dependent transcription termination (Hoyos et al., 2020). It is noteworthy that most of 3′UTR-derived sRNAs modulate the expression of proteins with functions related to the genes they originate from. Therefore, 3′UTRs could be considered as additional functional units complementing polycistronic operons. Among the several transcriptomic mapping analyses carried out so far, there are still numerous 3′UTR-derived sRNA candidates that need to be studied. Therefore, it is expected that the number and functions of these kind of post-transcriptional regulators will increase in the near future.

**Table 1:** Relevant 3′UTR-derived sRNAs characterized in bacteria.

**Species**	**mRNA**	**sRNA**	**Targets**	**Relevant characteristics**	**References**
**Gram-negative bacteria**					
**Type I 3′UTR-derived sRNAs**					
*Escherichia coli*	*cutC*	MicL	*lpp*	σ^E^-dependent, involved in membrane stress	Guo et al., 2014
*Pseudomonas aeruginosa*	*PA4570*	RsmW	RsmA	Sponge RNA that sequesters the RsmA protein, modulating biofilm formation	Miller et al., 2016
*Salmonella*	*dapB*	DapZ	*oppA* *dppA*	HilD-dependent, represses two ABC transporters	Chao et al., 2012
*Vibrio cholerae*	*vca0943*	MicX	*vc0972* *vc0620*	Processed by RNase E in a Hfq-dependent manner, regulates an outer membrane protein, and an ABC transporter	Davis and Waldor, 2007
**Type II 3′UTR-derived sRNAs**					
*Escherichia coli*	*malEFG*	MalH	*ompC* *ompA* *MicA*	Hfq-dependent, promotes accumulation of maltose transporters during transition growth phase	Iosub et al., 2020
	*sdhCDAB-sucABCD*	SdhX	*ackA* *fdoG* *katG*	Coordinates the expression of the TCA cycle and the acetate metabolism	De Mets et al., 2019; Miyakoshi et al., 2019
*Rhodobacter sphaeroides*	*RSP_0847*	SorX	*potA*	Inhibits a polyamine transporter to counteract oxidative stress	Peng et al., 2016
*Salmonella*	*cpxP*	CpxQ	*agp* *fimAICDHF* *nhaB* *skp-lpxD* *ydjN*	Hfq-dependent, targets extracytoplasmic proteins to alleviate inner membrane stress	Chao and Vogel, 2016
	*narK*	NarS	*nirC*	Hfq-dependent, involved in nitrate respiration homeostasis	Wang et al., 2020
	*raiA*	RaiZ	*hupA*	ProQ-dependent, represses translation of *hupA* mRNA	Smirnov et al., 2017
	*sdhCDAB-sucABCD*	SdhX	*ackA* *fumB* *yfbV*	Coordinates the expression of the TCA cycle and the acetate metabolism	Miyakoshi et al., 2019
	*gltIJKL*	SroC	GcvB	Sponge RNA, alleviates GcvB repression of amino acid transport, and metabolic genes	Miyakoshi et al., 2015a
*Vibrio cholerae*	*carAB*	CarZ	*carAB*	It negatively autoregulates the operon from which it is processed	Hoyos et al., 2020
	*fabB*	FarS	*fadE*	Hfq-dependent, regulates fatty acid metabolism	Huber et al., 2020
	*oppABCDF*	OppZ	*oppABCDF*	It negatively autoregulates the operon from which it is processed	Hoyos et al., 2020
**Gram-positive bacteria**					
**Type I 3′UTR-derived sRNAs**					
*Lactococcus lactis*	*argR*	ArgX	*arcC1*	Regulates the arginine metabolism	van der Meulen et al., 2019
**Type II 3′UTR-derived sRNAs**					
*Staphylococcus aureus*	*mntABC*	RsaC	*sodA*	Involved in the oxidative stress response and metal homeostasis	Lalaouna et al., 2019
*Streptomyces coelicolor*	*sodF*	s-SodF	*sodN*	Inhibits SodN, regulates superoxide dismutases expression depending on nutrient availability	Kim et al., 2014

### 3′UTRs That Target Other mRNAs in *Trans*

In some cases, the whole mRNA molecule can act as a regulatory RNA by itself and modulate the expression of other mRNAs in *trans*. This is the case of RNAIII from *S. aureus*. Asides from *cis*-regulating the δ-hemolysin translation through a 5′UTR-3′UTR interaction, it modulates in *trans* several mRNA targets that encode surface proteins and virulence factors at the post-transcriptional level (Balaban and Novick, 1995; Bronesky et al., 2016). For instance, the binding of RNAIII to the *spa, coa, sbi*, and *rot* mRNAs impairs their translation and it often leads to RNase III processing (Huntzinger et al., 2005; Boisset et al., 2007; Chevalier et al., 2010; Chabelskaya et al., 2014). However, RNAIII regulation is not always in the form of repression. The *mgrA* and *hla* mRNAs are positively regulated after interacting with RNAIII through stabilization of the mRNA in the former (Gupta et al., 2015) and activation of translation in the latter (Morfeldt et al., 1995). Overall, the duality of RNAIII as a regulator resides in its ability to repress surface proteins while promoting the expression of secreted virulence factors (Bronesky et al., 2016; Raina et al., 2018). In *L. monocytogenes*, the 3′UTR of the *hly* mRNA (listeriolysin O) base pairs with the 5′UTR of the *prsA2* mRNA (Ignatov et al., 2020). Both, listeriolysin O and PrsA2 are virulence factors necessary for *L. monoctyogenes* infection cycle (Alonzo and Freitag, 2010; Hamon et al., 2012). This mRNA-mRNA interaction prevents the degradation of the *prsA2* mRNA by RNase J1. When mutations are introduced in the *hly* 3′UTR or *prsA2* 5′UTR, the interaction between them is abolished resulting in reduced pathogenicity (Ignatov et al., 2020).

## Differential Evolution of 3′UTRs in Bacteria

The sequence of some 3′UTRs have been shown to be variable depending on the bacterial species. The *dapB* and *sdh* 3′UTRs, which produce the 3′UTR-derived sRNAs DapZ and SdhX, respectively, show nucleotide variations between different enterobacterial species, leading to functional variability (Chao et al., 2012; De Mets et al., 2019; Miyakoshi et al., 2019). For instance, SdhX represses different mRNA targets in *E. coli* and *Salmonella* through a slightly different seed sequence (Miyakoshi et al., 2019). In *S. aureus*, the *icaR* 3′UTR presents a sequence divergence when compared to other staphylococcal species (Ruiz de Los Mozos et al., 2013). In fact, the sequence downstream of *icaR* is completely different to its close relative, *S. epidermidis*. Although, the *S. epidermidis icaR* 3′UTR has a similar length to that of *S. aureus* (365 vs 391 nt, respectively), the UCCCC motif or an equivalent sequence to pair with the 5′UTR are lacking. Instead, the *S. epidermidis* IcaR 5′UTR is targeted by the IcaZ sRNA, which impairs the *icaR* mRNA translation. Interestingly, the IcaZ sRNA is encoded just downstream of the *S. epidermidis icaR* mRNA, indicating that not only the 3′UTRs are different but also the regions downstream of the transcriptional terminator (Lerch et al., 2019). Sequence variations found downstream of the *icaR* CDS could be explained by gene rearrangements that occurred when the *icaRADBC* operon was acquired by certain staphylococcal species (Menendez-Gil et al., 2020). Note that only 9 staphylococcal species encode the *ica* genes and that their genomic position varies from one another. In 4 of them the *icaR* gene appears to be replaced by other genes that encode DspB (hexosaminidase), a TetR-like regulator, or proteins of a two-component system that might be involved in controlling the PIA-PNAG exopolysaccharide production through different mechanisms. These gene rearrangements could have naturally led to the occurrence of different *icaR* mRNA chimeras among staphylococcal species and, therefore, explain the currently observed functional differences at the post-transcriptional level (Menendez-Gil et al., 2020). A recent genome-wide study carried out by our group revealed that variations on 3′UTR sequences were widespread. We found that most of the 3′UTRs from orthologous genes were not conserved among species of the genus *Staphylococcus* (Menendez-Gil et al., 2020). These 3′UTRs differed both in sequence and length. The mRNA sequence conservation was lost around the stop codon. When comparing close relative species, the nucleotide variations occurred through different processes, including gene rearrangements, local nucleotide changes and transposition of insertion sequences. Swapping the 3′UTR sequences produced changes in the mRNA and protein levels of conserved staphylococcal genes, suggesting the existence of different regulatory elements in each orthologous mRNA. Interestingly, this differential evolution applied to most of the mRNAs encoding orthologous proteins among species of *Enterobacteriaceae* family and *Bacillus* genus. Also, several previously described functional 3′UTRs, including *E. coli acnB, Y. pestis hmsT*, C. *glutamicum aceA*, and *B. subtilis hbs*, were not conserved among orthologous mRNAs of their close-relative species. This widespread 3′UTR variability might be responsible for creating different functional regulatory roles and, ultimately, bacterial diversity through the course of evolution, resembling the process of diversification of eukaryotic species (Menendez-Gil et al., 2020).

## Perspectives

Historically bacterial 3′UTRs have been undervalued, probably because the real boundaries of the transcript 3′ ends were missed and/or the analyses that looked for functional non-coding RNAs were biased by parameters such as sequence conservation. However, the examples presented in this MiniReview show that 3′UTRs contain a wide repertoire of diverse regulatory elements and they constitute a key regulatory layer to be considered when studying post-transcriptional regulation in bacteria. It is clear that 3′UTRs are involved in the modulation of relevant biological processes such as metabolism, iron homeostasis, biofilm formation and virulence, among others. Although a lack of sequence conservation was often associated with a lack of function, the differential evolution found within 3′UTRs from close-relative bacteria should help changing our overall perception about them. Considering that eukaryotic 3′UTRs have been utilized by evolution to create alternative regulatory pathways and, hence, contribute to species diversification, a similar phenomenon might be envisioned for bacterial 3′UTRs. Although few examples support this theory so far (Chao et al., 2012; Ruiz de Los Mozos et al., 2013; Lerch et al., 2019; Miyakoshi et al., 2019; Menendez-Gil et al., 2020), it is expected that, by studying the vast numbers of the recently identified long 3′UTRs and their orthologs, we will finally understand the biological relevance of 3′UTR mediated-regulatory mechanisms.

## Author Contributions

PM-G and AT-A contributed to the conception, contents, and writing of this manuscript. All authors contributed to the article and approved the submitted version.

## Conflict of Interest

The authors declare that the research was conducted in the absence of any commercial or financial relationships that could be construed as a potential conflict of interest.
